# Effect of PGPRs on the Rhizosphere Microbial Community Structure and Yield of Silage Maize in Saline–Alkaline Fields

**DOI:** 10.3390/ijms26168040

**Published:** 2025-08-20

**Authors:** Weisong Zhao, Shezeng Li, Wei Yang, Naqi Cui, Xiuyun Lu, Shaojing Mo, Qinggang Guo, Ping Ma

**Affiliations:** Key Laboratory of IPM on Crops in Northern Region of North China, Institute of Plant Protection, Hebei Academy of Agriculture and Forestry Sciences, Integrated Pest Management Innovation Centre of Hebei Province, Ministry of Agriculture and Rural Affairs of China, Baoding 071000, China; zhaoweisong1985@163.com (W.Z.); shezengli@163.com (S.L.); victor.02@163.com (W.Y.); cuinaqi15200@163.com (N.C.); luxiuyun03@163.com (X.L.); msjing1983@163.com (S.M.)

**Keywords:** PGPR, saline-alkaline soils, silage maize, soil nutrients, rhizosphere microbiome, microbial community structure

## Abstract

Plant Growth Promoting Rhizobacteria, PGPR, can protect plants against soil-borne diseases and abiotic stress conditions. The primary objective of this study was to evaluate the effects of different PGPRs (TF1, TF2, TF3, and TF4) on the rhizosphere microbial community of silage maize in a saline–alkaline field via Illumina MiSeq high-throughput sequencing technology. Results demonstrated that different PGPRs significantly increased the harvest density (by 21.31–45.16%), plant height (by 9.12–19.98%), stem diameter (by 30.07–45.78%), and biomass (by 33.20–65.36%) of silage maize, TF3 treatment significantly increased the fresh weight (by 32.50%), while the other treatments could increase the fresh weight but not significantly. Four microbial agents significantly reduced the contents of soil available phosphorus (AP), electrical conductivity (EC), and neutral phosphatase activity (NPA), while significantly increasing the contents of available potassium (AK), ammonium nitrogen (NH_4_^+^-N), nitrate nitrogen (NO_3_^−^-N), chitinase activity (ChtA), and urease activity (UA). Specifically, TF2 and TF3 treatments significantly decreased the soil pH value, while not for TF1 and TF4. Microbiome analysis showed that four microbial agents significantly increased the relative abundances of beneficial microorganisms, such as *Arthrobacter*, *Blastococcus*, *MNDI*, *Chaetomidium*, *Alternaria*, *Sarocladium*, *Acremonium*, and *Clonostachys*, and significantly decreased the relative abundances of *Gibberella* and *Fusarium*. Mental analysis showed that the soil bacterial community structure did not significantly correlate with soil biochemical properties, while the soil fungal community structure significantly and positively correlated with pH. Maize yield significantly and positively correlated with NH_4_^+^-N, OM, AP, EC, UA, ChtA, and NPA.

## 1. Introduction

Maize is an important food crop that is crucial to the development of the entire national economy in China. With the improvement of people’s living standards, there has been a significant shift in the demand for maize. Gradually, maize has transformed from a food crop to a feed crop [1]. In recent years, with the development of China’s animal husbandry and the adjustment of planting structure, as well as the implementation of the “grain to forage” policy, the planting area and scale of silage maize have been increasing rapidly [2]. The No. 1 Central Document in 2023 clearly proposed to “vigorously develop silage and accelerate the promotion of straw-based livestock breeding”, indicating that the silage maize industry has great development potential [3].

Soil salinization is a major global environmental and resource issue; due to climate change, the area of saline–alkaline soils is increasing year by year, and about 50% of the world’s arable land will be affected by salinity in 2050, which is one of the biggest global challenges that severely affects agricultural productivity and environmental sustainability [4,5,6]. In China, saline–alkaline soils cover a large area and are widely distributed. In recent years, the salinization trend of cultivated land in some areas has intensified with climate change. Therefore, it is of great significance to carry out a comprehensive renovation and utilization of saline-alkaline soil. The Heilonggang region is a typical saline–alkaline soil distribution area in the North China Plain. In the city of Cangzhou of Hebei Province, in particular, saline–alkaline soil is widespread. In some areas, the high groundwater level and high soil salt content have led to moderate to severe salinization, which severely restricts crop production and the healthy and sustainable development of local grass-based animal husbandry [7]. Therefore, research related to the improvement and utilization of saline–alkaline soil has increasingly drawn people’s attention. The application of PGPR in the improvement of saline–alkaline soil has become a research hotspot. They are characterized by being environmentally friendly, significantly improving the soil environment, enhancing soil fertility, promoting crop growth, and supporting the sustainable utilization of saline–alkaline soil [8,9]. In addition, the application of microbial agents has been shown to increase soil enzyme activity and enhance crop yield [10,11]. However, the application effects of microbial agents are susceptible to influence by external environmental conditions, and the high cost of microbial agents limits their application scope to a certain extent [10].

Soil microorganisms are considered one of the important indicators for evaluating soil quality [12]. The application of microbial agents not only changes the soil microbial community and improves soil enzyme activity but also reduces soil salinity and pH and ameliorates soil nutrients, which can also enhance a plant’s salt tolerance or reduce the occurrence of crop diseases and pests, ultimately leading to an increased yield [13,14,15,16,17,18].

In response to the issues of a low seedling emergence rate and low yield in silage maize cultivation in the saline–alkaline soils of Cangzhou in Hebei Province, four self-developed microbial agents were used in the study, and the effects of different microbial agents on the seedling protection and yield increase in silage maize in saline–alkaline soil were systematically evaluated, as well as their impact on the rhizosphere microbial community by high-throughput sequencing technology. This study aims to reveal the driving factors of rhizosphere microbial community changes mediated by microbial agents, elucidate the microbial mechanisms by which microbial agents enhance salt tolerance and seedling establishment in silage maize, and also provide a theoretical basis for the application of microbial agents in the cultivation of silage maize in saline–alkaline soils.

## 2. Results

### 2.1. Effects of Different PGPRs on the Growth Promotion of Silage Maize

PGPR inoculation significantly influenced the growth parameters and biomass of silage maize (*p* < 0.05) (Table 1). Compared with the blank control, the plant density of treatments TF1, TF2, TF3, and TF4 increased significantly by 23.18%, 21.32%, 22.33%, and 43.03%, respectively. The plant height of these treatments increased significantly by 19.46%, 19.98%, 18.27%, and 9.12%, respectively. The fresh weight per plant of TF3 treatment increased significantly by 32.50%, while the fresh weight per plant of TF1, TF2, and TF4 treatments increased by 13.75%, 17.5%, and 21.25%, respectively, but showed no significant difference compared with the blank control. The stem diameter of treatments TF1, TF2, TF3, and TF4 increased significantly by 45.78%, 33.23%, 34.51%, and 30.07%, respectively. The biomass of treatments with PGPR significantly increased by 33.19%, 38.25%, 54.04%, and 65.34%, respectively (Table 1).

### 2.2. Effects of Different PGPRs on Soil Nutrients and Enzyme Activities

The results in terms of soil nutrients indicate that the treatments with TF1, TF2, and TF3 increased the organic matter content by 9.04%, 2.26%, and 18.64%, respectively, yet there was no significant difference compared with the blank control (Table 2). In contrast, the organic matter content in the TF4 treatment was significantly increased by 29.94% (*p* < 0.05). Meanwhile, the application of microbial agents significantly increased the contents of soil NO_3_^−^-N, NH_4_^+^-N, and AK (*p* < 0.05). In contrast, compared with the blank control, the application of PGPRs significantly reduced the AP content and EC in the soil. Specifically, the AP contents in TF1, TF2, TF3, and TF4 treatments were significantly reduced by 81.47%, 74.50%, 75.23%, and 88.62%, respectively, and ECs were also significantly reduced by 32.93%, 39.72%, 17.43%, and 26.50%, respectively. There was no significant difference in soil pH between the TF1 and TF4 treatments and the blank control, while the soil pH in the TF2 and TF3 treatments were significantly reduced by 4.70% and 2.96%, respectively. In terms of soil enzyme activities, different PGPRs significantly increased chitinase and urease activities. Specifically, the chitinase activities were increased by 6.55%, 23.08%, 8.83%, and 17.08%, respectively, and the urease activities were increased by 8.54%, 17.89%, 19.83%, and 26.27%, respectively. Moreover, microbial agents significantly reduced neutral phosphatase activities, with no significant difference observed in invertase enzyme activities (Table 2).

### 2.3. Alpha Diversity, Bacterial, and Fungal Community Structure Analysis Under Different PGPRs

**Figure 1:**
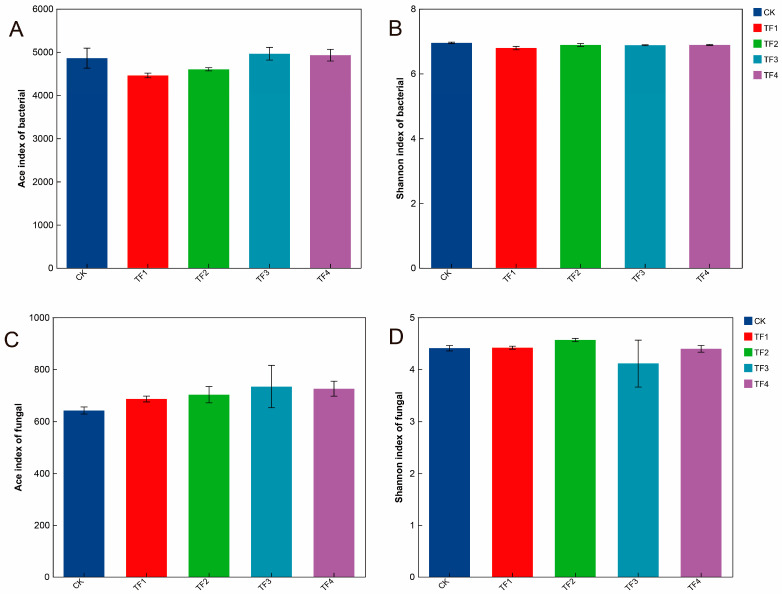
Effect of microbial agents on alpha diversity index of soil microbial community in rhizosphere. (**A**,**B**) Alpha diversity of bacterial community, (**C**,**D**) alpha diversity of fungal community. CK represents treatment with blank control, TF1 represents treatment with *B. subtilis* NCD-2 + *B. amyloliquefaciens* Ba-1, TF2 represents treatment with *B. subtilis* HMB26553 and *B. amyloliquefaciens* PHODG36, TF3 represents treatment with *B. subtilis* WHN-121 and *B. amyloliquefaciens* G35, and TF4 represents treatment with *B. velezensis* B31.

ANOVA indicated that the microbial agents did not significantly affect the Ace and Shannon indices of soil bacteria and fungi, compared with the blank control treatment. Additionally, no significant differences were observed among different microbial agents (Figure 1).

**Figure 2:**
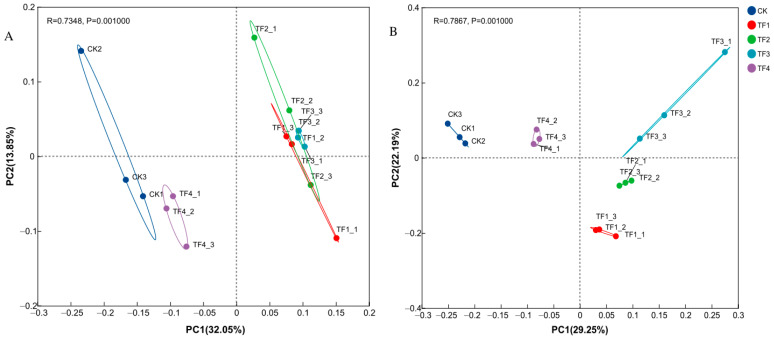
Principal component analysis of bacterial (**A**) and fungal (**B**) community structure in rhizosphere under different treatments. CK represents treatment with blank control, TF1 represents treatment with *B. subtilis* NCD-2 + *B. amyloliquefaciens* Ba-1, TF2 represents treatment with *B. subtilis* HMB26553 and *B. amyloliquefaciens* PHODG36, TF3 represents treatment with *B. subtilis* WHN-121 and *B. amyloliquefaciens* G35, and TF4 represents treatment with *B. velezensis* B31.

In terms of bacteria, the cumulative explained variance of the two principal components (PC) of the rhizosphere bacterial communities at the OTU level was 45.90%, with the first principal component (PC1) accounting for 32.05% and the second principal component (PC2) accounting for 13.85%. The bacterial communities of the control (CK) and four microbial agent treatments (TF1, TF2, TF3, and TF4) showed no overlap, indicating that each microbial agent altered the bacterial community structure. Specifically, TF1, TF2, and TF3 were positioned in different quadrants from TF4, indicating that the bacterial community structure of TF4 was distinctly different from the other three treatments. Meanwhile, the bacterial communities of TF1, TF2, and TF3 exhibited some overlap, suggesting similar community structures among these treatments (Figure 2A). In terms of fungi, the cumulative explained variance of the two principal components (PC) of the rhizosphere fungal communities at the OTU level was 51.44%, with the first principal component (PC1) accounting for 29.25% and the second principal component (PC2) accounting for 22.19%. The fungal communities of the control (CK) and four microbial agent treatments (TF1, TF2, TF3, and TF4) showed no overlap, indicating that each microbial agent altered the fungal community structure. The fungal communities of four microbial agent treatments were distinctly separated, suggesting that the fungal community structures of TF1, TF2, TF3, and TF4 were all different from each other (Figure 2B).

### 2.4. Effects of PGPRs on the Composition of Rhizosphere Soil Microbial Communities at the Phylum Level

**Figure 3:**
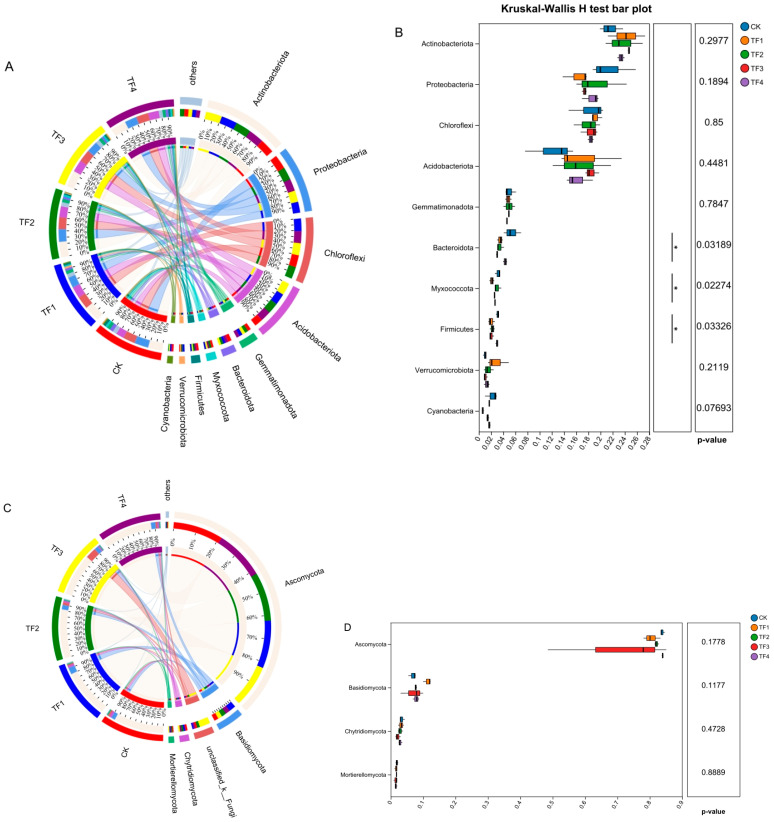
Comparison of relative abundance of dominant bacterial and fungal communities under different treatments at the phylum level. (**A**) Relative abundance of dominant bacterial community, (**B**) significant analysis of bacterial community abundance at the phylum level, (**C**) relative abundance of dominant fungal community, and (**D**) significant analysis of fungal community abundance at the phylum level (* *p* < 0.05). CK represents treatment with blank control, TF1 represents treatment with *B. subtilis* NCD-2 + *B. amyloliquefaciens* Ba-1, TF2 represents treatment with *B. subtilis* HMB26553 and *B. amyloliquefaciens* PHODG36, TF3 represents treatment with *B. subtilis* WHN-121 and *B. amyloliquefaciens* G35, and TF4 represents treatment with *B. velezensis* B31.

At the phylum level, the study of bacterial communities in saline–alkaline soils revealed that the dominant phyla across different treatments were Actinobacteriota, Proteobacteria, Chloroflexi, Acidobacteriota, Gemmatimonadota, Bacteroidota, Myxococcota, Firmicutes, Verrucomicrobiota, and Cyanobacteria, and their relative abundances ranged from 21.62% to 24.67%, 16.33% to 21.50%, 17.98% to 19.23%, 12.24% to 18.47%, 4.62% to 5.04%, 2.99% to 5.40%, 2.14% to 3.16%, 2.03% to 3.11%, 1.02% to 2.85%, and 0.66–2.22%, respectively. The average relative abundance of these dominant phyla accounted for over 93% of the total bacterial communities (Figure 3A). A Kruskal–Wallis analysis indicated the relative abundances of Bacteroidota, Myxococcota, and Firmicutes in four microbial agent treatments significantly decreased. In contrast, the relative abundances of Actinobacteriota, Acidobacteriota, and Verrucomicrobiota increased but not significantly in four microbial agent treatments compared with the blank control (Figure 3B).

For fungi, the results indicated that the dominant fungal phyla in saline–alkaline soils were Ascomycota, Basidiomycota, unclassified phyla, Chytridiomycota, and Mortierellomycota, and their relative abundances ranged from 70.51% to 83.89%, 6.79% to 11.47%, 2.05% to 18.05%, 2.17% to 3.46%, and 1.53% to 1.88%. Except for the treatment with microbial agent TF3, the average relative abundances of these dominant phyla accounted for over 98% in the other treatments (Figure 3C). A Kruskal–Wallis analysis indicated the relative abundances of Ascomycota, Chytridiomycota, and Mortierellomycota showed a downward trend, while that for Basidiomycota increased in the four microbial agent treatments. However, no significant differences were observed in these phyla, compared with the blank control (Figure 3D).

### 2.5. Effects of PGPRs on the Composition of Rhizosphere Soil Microbial Communities at the Genus Level

**Figure 4:**
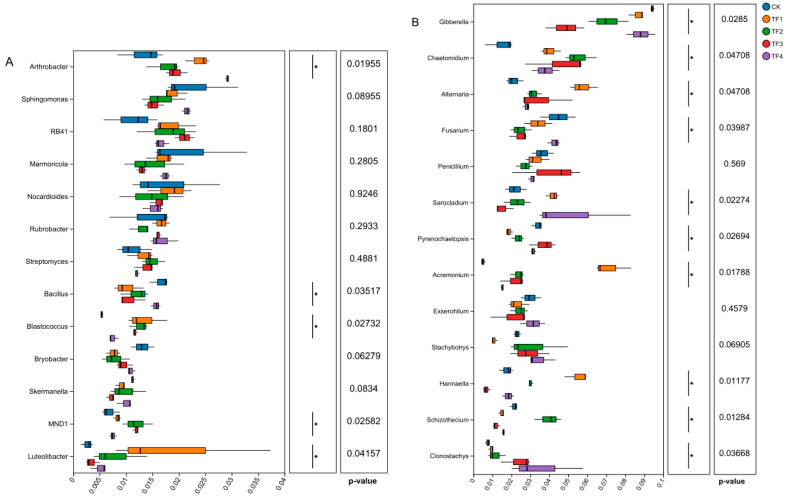
Comparison of relative abundance of dominant bacterial and fungal communities under different treatments at the genus level. (**A**) Significant analysis of bacterial community abundance at the genus level (* *p* < 0.05). (**B**) Significant analysis of fungal community abundance at the genus level (* *p* < 0.05). CK represents treatment with blank control, TF1 represents treatment with *B. subtilis* NCD-2 + *B. amyloliquefaciens* Ba-1, TF2 represents treatment with *B. subtilis* HMB26553 and *B. amyloliquefaciens* PHODG36, TF3 represents treatment with *B. subtilis* WHN-121 and *B. amyloliquefaciens* G35, and TF4 represents treatment with *B. velezensis* B31.

The results of bacterial community composition at the genus level indicated that the dominant and identifiable genera with relative abundances greater than 1% in the saline–alkaline soils were *Arthrobacter*, *Sphingomonas*, *RB41*, *Marmoricola*, *Nocardioides*, *Rubrobacter*, *Streptomyces*, *Bacillus*, *Blastococcus*, *Bryobacter*, *Skermanella*, *MND1*, and *Luteolibacter*. A Kruskal–Wallis analysis indicated the relative abundances of *Arthrobacter* increased significantly by 78.62%, 32.13%, 45.01%, and 118.25% in the TF1, TF2, TF3, and TF4 treatments, respectively; the relative abundances of *Blastococcus* increased significantly by 148.90%, 135.43%, 117.42%, and 40.12% in the TF1, TF2, TF3, and TF4 treatments, respectively; and the relative abundances of *MND1* increased significantly by 22.32%, 74.11%, 73.53%, and 9.55% in the TF1, TF2, TF3, and TF4 treatments, respectively, compared with the blank control treatment. In contrast, the microbial agent treatments significantly reduced the relative abundances of *Bacillus* and *Luteolibacter*. Meanwhile, the relative abundances of *Sphingomonas*, *Marmoricola*, *Bryobacter*, and *Skermanella* showed a downward trend in different microbial agent treatments, while that of *Streptomyces* and *RB41* exhibited an upward trend, although no significant differences were observed compared with the blank control (Figure 4A).

On the other hand, the results of fungal community composition at the genus level indicated that the top 13 dominant and identifiable genera with relative abundances greater than 1% in saline–alkaline soils were *Gibberella*, *Chaetomidium*, *Alternaria*, *Fusarium*, *Penicillium*, *Sarocladium*, *Pyrenochaetopsis*, *Acremonium*, *Exserohilum*, *Stachybotrys*, *Hannaella*, *Schizothecium*, and *Clonostachys*. A Kruskal–Wallis analysis indicated the relative abundances of *Gibberella* decreased significantly by 7.95%, 24.61%, 48.18%, and 6.86% under four microbial agent treatments, respectively; the relative abundances of *Fusarium* also decreased significantly by 23.70%, 44.69%, 45.06%, and 5.44%, respectively, compared with the blank control. For *Schizothecium*, the relative abundances decreased significantly by 32.19%, 45.77%, and 29.03% under TF1, TF3, and TF4 treatments. In addition, microbial agent treatments significantly increased the relative abundances of *Chaetomidium*, *Alternaria*, *Sarocladium*, *Acremonium*, and *Clonostachys*. Meanwhile, the relative abundances of *Exserohilum* decreased by 22.31%, 18.97%, and 30.54% under TF1, TF2, and TF3 treatments, while that of *Stachybotrys* increased by 33.15%, 22.71%, and 50.25% under TF2, TF3, and TF4 treatments. The relative abundances of *Hannaella* were differentially affected under four microbial agent treatments.

### 2.6. Relationships Between Soil Microbial Community, Yield, and Soil Biochemical Properties

**Figure 5:**
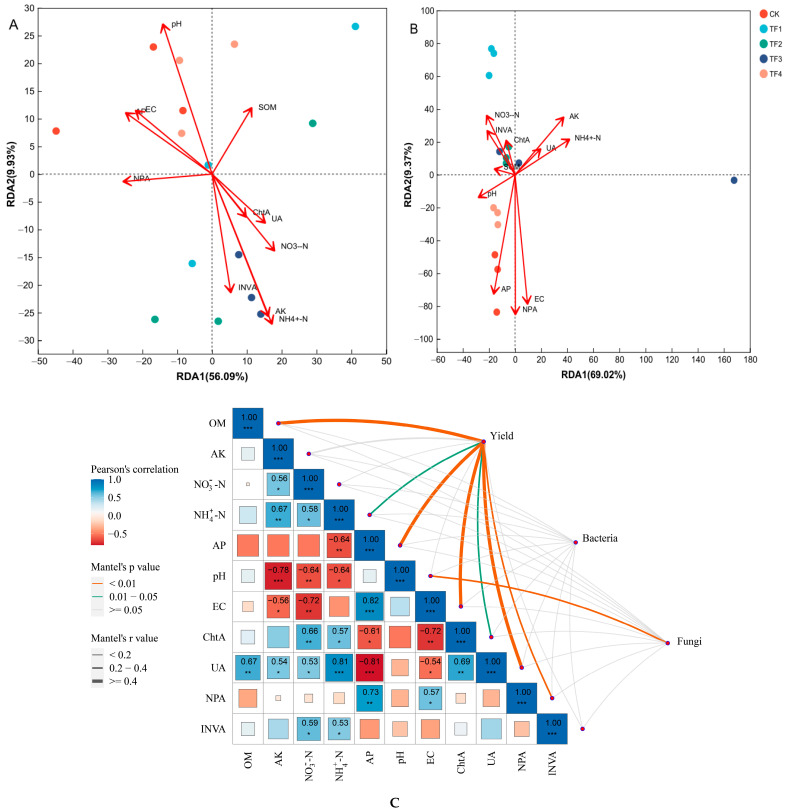
Relationships between soil microbial community, yield, and soil biochemical properties. (**A**) RDA of rhizosphere bacterial community composition with soil biochemical properties, (**B**) RDA of rhizosphere fungal community composition with soil biochemical properties, and (**C**) Pearson’s correlation analysis between soil microbial community structure at the phylum level, yield, and soil biochemical properties. CK represents treatment with blank control, TF1 represents treatment with *B. subtilis* NCD-2 + *B. amyloliquefaciens* Ba-1, TF2 represents treatment with *B. subtilis* HMB26553 and *B. amyloliquefaciens* PHODG36, TF3 represents treatment with *B. subtilis* WHN-121 and *B. amyloliquefaciens* G35, and TF4 represents treatment with *B. velezensis* B31. * represents 0.01 < *p* < 0.05, ** represents 0.001 < *p* < 0.01, *** represents *p* < 0.001, respectively.

A redundancy analysis (RDA) was used to examine the relationships between soil bacterial and fungal communities and soil biochemical properties (Figure 5A,B). The first two RDA components (RDA1 and RDA2) explained 56.09% and 9.93% of the total variance in the bacterial community, and the cumulative contribution rate could reach 66.02% of the total variation (Figure 5A). The r^2^ and *p*-values were determined to elucidate the significance of the relationships between soil biochemical properties and microbial communities’ compositions. The pH (r^2^ = 0.4984, *p* = 0.017), NH_4_^+^-N (r^2^ = 0.5497, *p* = 0.011), and AK (r^2^ = 0.4904, *p* = 0.015) significantly correlated with the bacterial communities under the microbial agent treatments. Among them, the bacterial communities in the blank control and TF4 treatment showed a positive correlation with pH and EC, while the bacterial communities in the other microbial agent treatments had a positive correlation with NH_4_^+^-N and AK.

For fungi, RDA1 and RDA2 could explain 69.02% and 9.37% of the variation, and the cumulative contribution rate could reach 78.39% of the total variation (Figure 5B). The AP (r^2^ = 0.4666, *p* = 0.019), EC (r^2^ = 0.5206, *p* = 0.01) and NPA (r^2^ = 0.6053, *p* = 0.004) significantly influenced the fungal communities under the microbial agent treatments. Among them, the fungal communities in the blank control and TF4 treatment showed a significant positive correlation with AP, EC, and NPA, while the fungal communities in the other microbial agent treatments had a significant negative correlation with AP, EC and NPA. In addition, the fungal communities treated with TF1, TF2, and TF3 microbial agents showed positive correlations with NH_4_^+^-N, AK, and NO_3_^−^-N, but these correlations were not statistically significant.

To further explore the effect of microbial community structure on the yield and soil biochemical properties, we took the mantel analysis to establish a link (Figure 5C). The results showed that the soil bacterial community structure was not significantly correlated with soil biochemical properties, while the soil fungal community structure was significantly and positively correlated with the pH (*p* < 0.05). In addition, yield was significantly and positively correlated with NH_4_^+^-N and ChtA (*p* < 0.05), and highly significantly and positively correlated with OM, AP, EC, UA, and NPA (*p* < 0.01).

## 3. Discussion

Saline–alkaline stress threatens the plant yield, protein synthesis, photosynthesis, and energy metabolism, but it also inevitably restricts agricultural productivity [19]. Our results indicate that the application of microbial agents could enhance the emergence and seedling retention of silage maize in saline–alkaline soils and increase plant fresh weight and biomass. This study revealed the beneficial effects of microbial agents on plant growth in saline–alkaline soils, which are consistent with many previous studies showing that microbial agents can improve plant growth performance, nutrient levels, and yield [20,21,22,23,24]. Meanwhile, the possible reason that the application of microbial agents increases crop yield in saline–alkaline soils is that the *Bacillus* species can secrete organic acids, thereby reducing soil alkalinity [25,26,27]. Previous studies have shown that beneficial microorganisms promote plant growth by secreting plant hormones, solubilizing phosphorus, fixing nitrogen, and enhancing plant nutrient uptake [28,29]. *Pseudomonas* and *Bacillus*-based microbial agents enhance the absorption of trace elements such as iron, manganese, zinc, and copper in maize plants, thereby increasing photosynthesis, carbohydrate metabolism, and the formation of plant hormones and chlorophyll [29]. In addition, beneficial microorganisms such as *Serratia liquefaciens* can induce systemic resistance in plants and promote plant growth [30]. However, the physiological and biochemical mechanisms by which microbial agents significantly increase the yield of silage maize in this study, as well as whether they have an impact on the quality of silage maize, remain to be further investigated.

Saline–alkaline soils with a high salt content and extreme pH levels have an adverse impact on the availability of soil nutrients and the activities of soil enzymes, which can disrupt soil nutrient cycling, reduce microbial activity, and limit the absorption of essential nutrients by plants [31]. Organic matter (OM), mineralizable nitrogen (including NO_3_^−^-N and NH_4_^+^-N), available phosphorus (AP), and available potassium (AK) in the soil play important roles in plant growth and development, the absorption of soil mineral elements, and the stability of the soil environment, which are also key indicators for assessing soil fertility [32]. Our research findings indicate that the application of microbial agents TF1, TF2, TF3, and TF4 significantly increased the content of AK in the rhizosphere of silage maize while decreasing the contents of AP. We speculate that these different microbial agents promote silage maize growth by enhancing the bioavailability of AK. In terms of AP, a possible reason for the decline in soil AP is that the application of microbial agents enhances plant P absorption, increasing the AP content within plants and thereby leading to a reduction in soil AP content. Therefore, the content of AP in plants requires further verification. Meanwhile, it was found that application of microbial agents significantly increased the contents of NO_3_^−^-N and NH_4_^+^-N in soil, which suggests that microbial agents promote soil nutrient mineralization, thereby enhancing plant nutrient uptake and utilization. The results of this study showed that four microbial agent treatments significantly increased the contents of NO_3_^−^-N and NH_4_^+^-N, while the content of OM showed an increasing trend but not significantly. It was speculated that microbial agents may also promote the growth of silage maize by enhancing the plant utilization of NO_3_^−^-N, NH_4_^+^-N, and OM.

Microbial agents can activate nutrients in the soil, converting them into forms that are available for plant uptake, thereby exerting growth-promoting effect on plants [33,34]. After spraying the lactic acid bacteria compound microbial agents on tomatoes planted in saline–alkaline soil, the contents of NO_3_^−^-N, NH_4_^+^-N, and AP in the soil increased significantly [33]. The application of *Bacillus megaterium* can improve the bioavailability of soil phosphorus and potassium, which is attributed to solubilizing phosphorus and enhancing the availability of potassium, thereby promoting plant growth and nutrient uptake [34]. *Bacillus* exhibited plant growth-promoting properties relevant to P dynamics [35] and N fixation [36], including phosphate solubilization and mineralization, production of indole-3-acetic acid (IAA)-like molecules [37], siderophores [38], exopolysaccharides [36], biofilms [36], and phosphatases [36]. However, the mechanisms by which four microbial agents increase nutrient content in rhizosphere soil will be studied in the future.

In addition, previous studies have shown that the application of microbial agents can reduce soil salt content and increase the soil desalination rate [25,39]. The results of this study indicated that the application of microbial agents significantly reduced soil EC, with a reduction range of 17.43% to 39.72%. Among four different microbial agent treatments, TF2 achieved the highest reduction. One possible reason is that beneficial microorganisms produce polysaccharides and mucilage during their activities, forming soil binders, which affect the soil aggregate structure and reduce bulk density and non-capillary pores in the soil, thus accelerating soil salt leaching and reducing soil salt content [39,40]. The application of microbial agents can reduce the total salinity of soil and the contents of Na^+^, Ca^2+^, and K^+^ [41]. Pang et al. [40] found that microbial agents had no significant effect on the alfalfa biomass under non-saline and mild saline stress conditions. However, microbial agents significantly increased the alfalfa biomass under moderate saline stress, which may be attributed to the ability of microbial agents to recruit certain beneficial microorganisms that produce large amounts of polysaccharides and mucilage, reducing soil bulk density and surface soil salinity, thus verifying the viewpoints of previous scholars [39]. Studies have shown that the application of composite microbial agents can significantly reduce soil EC [25,42,43,44].

The results of this study indicated that treatment with microbial agents has varying effects on soil pH. There is no significant difference in soil pH between the treatments with microbial agents TF1 and TF4 and the control. However, the soil pH in the treatments with microbial agents TF2 and TF3 decreased significantly by 0.35 and 0.22 units, respectively. Microbial agents enhance the activity of phosphatase in the plant rhizosphere and promote the secretion of organic acids, which reduce the soil pH [33,45]. Hou et al. [33] found that the application of lactic acid bacteria consortia significantly decreased the soil pH by 0.42 units, and they proposed that lactic acid bacteria can lower soil pH through the secretion of organic acids and other metabolic products. Pang et al. [42] demonstrated that the application of composite microbial agents reduced soil pH by 0.92 units. Zhang et al. [14] reported that two different microbial agents significantly reduced soil pH, with reductions of 0.20 and 0.14 units, respectively. Wang et al. [46] demonstrated that the application of the *B. amyloliquefaciens* agent significantly reduced the soil pH in the rhizosphere of sunflowers by 0.17 units. However, there was no significant difference in soil pH after application of the *B. tequilensis* agent compared to the blank control. Microbial agents significantly reduced soil phosphatase activity (Table 2), which is contrary to the results reported by Guo [45]. Therefore, we speculate that the ability of microbial agents TF2 and TF3 to secrete organic acids is greater than that of TF1 and TF4, a hypothesis that should be further studied.

Soil microbial diversity is an important indicator for maintaining the functions and sustainability of agricultural ecosystems [47]. The application of microbial agents can enhance the abundance of rhizosphere microorganisms, alter microbial community composition and functional diversity, and maintain the balance of plant rhizosphere microbiota, which ultimately leads to improved soil fertility and promotes healthy crop growth [48]. From Figure 2, we obtained that the microbial community structure of the TF4 treatment was unlike other microbial agents; one possible reason is that the TF4 agent (single strain) may occupy distinct ecological niches vs. the other microbial agents (multi-strain consortia). Scholars had different views on the impact of microbial agents on soil microbial diversity [14,24,25,49,50]. The results of this study indicate that the application of four different microbial agents did not significantly affect soil microbial diversity; similar viewpoints were obtained as those of Griffiyhs et al. [39], Wang et al. [51], and Zhang et al. [14], wherein the possible reason could be that the microorganisms had not yet exerted their influence on environmental changes, as microbial agents require a certain period of adaptation time to alter the environment, and changes in microbial diversity need a specific time frame. However, some studies indicate that microbial agents can significantly alter soil microbial community structure and enhance microbial diversity in rhizosphere soils [25,52]. Higher microbial community richness is associated with functional uniqueness, indicating that microbial taxa with different functions possess high diversity, which is conducive to maintaining a healthier agricultural ecosystem [52]. In addition, other research suggests that microbial agents could increase the richness and diversity of the soil bacterial community and enrich bacteria beneficial to plant growth, but they could also reduce the richness and diversity of the soil fungal community [24,49,50].

Proteobacteria can decompose complex organic matter in plant residues, releasing nutrients for plant growth. Our study indicated that Proteobacteria exhibited a higher relative abundance in the rhizosphere soil of silage maize treated with microbial agents, which is similar to the findings of Ahsan [24]. The genera *Streptomyces* and *Arthrobacter* are important members of the phylum Actinobacteria. They are not only decomposers of organic matter but also capable of suppressing soil-borne pathogens. The application of microbial agents increases the relative abundances of these genera in the study, and previous studies are consistent with this finding [53,54]. Acidobacteriota plays an important role in the pH balance of soil and the decomposition of organic matter in saline–alkaline soils [55]. Our study indicated that Acidobacteriota exhibited a higher relative abundance in the rhizosphere soil of silage maize treated with microbial agents, which is contrary to the findings of Ai et al. [56] and Wu et al. [25]. The possible reason is that the response of soil microorganisms in saline–alkaline soils to microbial agents largely depends on local environmental factors, which often vary among study sites. Bacteroidota and Myxococcota are known for their ability to decompose plant residues and organic matter in the soil, thereby enhancing soil fertility. However, the results of this study indicate that the relative abundances of Bacteroidota and Myxococcota showed a significant decreased in the rhizosphere soil of silage maize treated with microbial agents. Moreover, based on the higher relative abundances of Proteobacteria and Acidobacteriota compared to Bacteroidota and Myxococcota, we speculate that in this saline–alkaline soil region, the application of microbial agents has a greater impact on Proteobacteria and Acidobacteriota than on Bacteroidota and Myxococcota. In addition, we found that the application of microbial agents decreased the relative abundances of most dominant fungal phyla but increased the relative abundance of Basidiomycota. It was suggested that the microbial agent treatments improved the health of the rhizosphere soil of silage maize, which is consistent with the view of Yuan et al. [57].

In this study, the application of four different microbial agents significantly increased the relative abundances of beneficial biocontrol microorganisms in rhizosphere soils, including *Arthrobacter* [28,58,59], *Blastococcus* [60], *Chaetomidium* [61], *Alternaria* [62,63], *Sarocladium* [64,65], *Acremonium* [66,67], and *Clonostachys* [68,69]. Conversely, it significantly decreased the relative abundances of potential plant pathogenic fungi, such as *Gibberella* and *Fusarium*. This finding is consistent with previous research that microbial agents could suppress potential plant pathogens and promote beneficial microbial communities [22,24,70]. Specific functional microorganisms in the microbial community can enhance the yield and quality of crops by increasing the availability of certain soil nutrients and promoting the absorption of nutrients by roots [71]. Wu et al. [25] demonstrated that *Gemmatimonadota* and *Verrucomicrobiota* could influence the nutrient utilization efficiency in soils by regulating enzyme activities, thereby exerting a growth-promoting effect on sweet sorghum. Qi et al. [70] demonstrated that microbial agents significantly promoted the growth of vegetables, altering the structure and function of the rhizosphere soil microbial community. Specifically, the relative abundances of beneficial microorganisms, including *Nitrospira*, *Nitrosomonas*, *Lysobacter*, and *Bacillus*, were increased, while the relative abundances of plant pathogenic fungi, such as *Fusarium* and *Alternaria*, were significantly reduced. It was demonstrated that the *B. megaterium* microbial agent promotes the growth of lettuce seedlings, while the microbial agent had no significant impact on the composition of dominant bacterial phyla in the soil but significantly increased the relative abundances of beneficial genera *Sphingomonas* and *Lysobacter* and reduced the relative abundance of potential pathogens [22]. Moreover, it was found that application of *B. methylotrophicus* did not elevate the abundance of *Bacillus* to become a dominant group in soil, with a possible reason being that *Bacillus* did not effectively colonize the soil, but this treatment activated the relative abundance of the dominant groups *Massilia* and *Streptomyces* [72]. It was also suggested that the disease-suppressive and growth-promoting effects of *B. megaterium* on cucumber plants might be due to the significant increase in the relative abundances of *Nocardioides* and *Streptomyces* [34].

## 4. Materials and Methods

### 4.1. Field Site Description

The field site is located in Haixing County, Cangzhou City, Hebei Province. It belongs to the warm-temperate, semi-humid monsoon climate zone. The main soil types in the field experimental site are primarily medium loamy and heavy loamy saline fluvo-aquic soil. The trial period was conducted from late June to early October 2023.

### 4.2. Experimental Description

We established the silage maize cultivation experiment in the Shengnong Agricultural Machinery Specialized Cooperative, in Xiangfang Township, Haixing County, Cangzhou City, Hebei Province, China (E 117°40′48′′, N 38°06′00′′). The planting system of this experimental field is a wheat–corn rotation. Four different microbial agents (TF1, TF2, TF3, and TF4) were provided by the Institute of Plant Protection, Hebei Academy of Agriculture and Forestry Sciences. Among these agents, the ingredients of TF1 included highly active strains of *Bacillus subtilis* NCD-2 and *B. amyloliquefaciens* Ba-1; the total effective number of viable bacteria is 3.0 billion CFU/mL. The ingredients of TF2 included active strains of *B. subtilis* HMB26553 and *B. amyloliquefaciens* PHODG36; the total effective number of viable bacteria is 4.5 billion CFU/mL. The ingredients of TF3 included active strains of *B. subtilis* WHN-121 and *B. amyloliquefaciens* G35; the total effective number of viable bacteria is 3.0 billion CFU/mL. The ingredient of TF4 included active strains of *B. velezensis*; the total effective number of viable bacteria is 5.0 billion CFU/mL. Microbial agents were prepared as seed-coating agents (Aqueous Solution, AS). In order to make microbial agents adhere to seeds evenly, the seeds and the agents were mixed on plastic film in a certain proportion (seeds:agents = 1:30, *w*/*v*) and stirred, and seeds were not coated with agents (representing CK). All seeds were planted on 30 June 2023. The silage maize cultivar was Zhengdan No. 958. Silage maize seeds were sown with a row spacing of 60 cm and a plant spacing of 25 cm, with approximately 4000 to 4500 plants per 667 m^2^, averaging 4250 plants. Each treatment had three sets of replicates for a total of n = 15 plots, and each plot had an area of 1/3 hm^2^.

### 4.3. Plant Analysis

During the silage maize harvest period (on 30 September 2023), three points were randomly selected for each treatment. Ten consecutive silage corn plants were chosen at each point to form one replicate. The plant spacing, plant height, stem diameter, and fresh weight of these plants were measured.

To calculate the theoretical biomass, (kg/hm^2^) = density (plants/hm^2^) × average fresh weight per plant (kg/plant), where density (plants/hm^2^) = (average plant spacing × row spacing/10,000) × 667 × 15.

### 4.4. Soil Sample Collection

Soil samples from each replicate were collected at the harvest period of silage maize (on 30 September 2023). Sampling points were located in each treatment; each sampling point randomly selected ten plants of silage maize. The entire root system of each plant in the 0–20 cm soil layer was dug out by spade, vigorously shaken to remove large soil particles loosely bound to the root, then the remaining particles closely attached to the root were collected as a rhizosphere soil sample [73]. Each ten rhizosphere samples were one biological replicate in each plot, and each plot sampled three replications. The collected soil from the five sampling points was mixed to generate a composite soil sample per plot. All soil samples were stored in iceboxes and immediately taken to the laboratory. There, each sample was divided into two parts: one portion of samples were sieved for 2.0 mm and stored at 80 °C for microbiomics analyses. The other soil samples were prepared for soil nutrients analyses and enzymatic activity.

### 4.5. Soil Chemical Analysis

The pH value was determined in a soil as follows: water suspension (1:5) using a pH meter (PHS-25, INESA, Shanghai, China) [74]; the electrical conductivity (EC) was determined in a soil as follows: water suspension (1:5) using a digital conductometer (DDSJ-308A, INESA, China) [75]. The available potassium (AK) was extracted with 1.0 M ammonium acetate, organic matter (OM) was determined with the potassium dichromate-ferrous sulfate titration method, and NO_3_^−^-N and NH_4_^+^-N were extracted with 2.0 M KCl and measured via ultraviolet spectrophotometer [76]. Available phosphorus (AP) was extracted by sodium bicarbonate and quantified with a spectrophotometer.

### 4.6. Soil Enzyme Assays

Soil urease activity (UA), invertase activity (INVA), chitinase activity (ChtA), and neutral phosphatase activity (NPA) were determined using the responding soil enzyme activity assay kit (No. BC0120, BC4040, BC1935, and BC0460) purchased from Solarbio Co., Ltd. (Beijing, China) as per the manufacturer’s protocol. UA was expressed as products (NH_3_^−^-N) per gram of dry weight soil per incubation time (24 h). INVA was expressed as the amount of reducing sugar produced per gram of soil per day at 37 °C. ChtA was expressed as the amount of N-acetylglucosamine produced by chitin decomposition per gram of soil per day at 37 °C. NPA was expressed as the amount of phenol produced per gram of soil per day at 37 °C.

### 4.7. Soil DNA Extraction, Illumina MiSeq Sequencing, and Bioinformatics Analysis

The extraction of DNA from rhizosphere soil was carried out by the instruction manual of the FastDNATM SPIN Kit (MP Biomedicals, Solon, OH, USA). A Drop 2000 spectrophotometer was used to estimate the concentration and purity of soil DNA. The extracted DNA was stored at −20 °C prior to further analyses. The investigation of bacterial and fungal communities involved using paired-end amplicon sequencing of the 16S rRNA gene and the ITS region of fungal ribosomal DNA. PCR amplification of the bacterial 16S rRNA was performed at the V3/V4 region with primers 338F (5′-ACTCCTACGGGAGGCAGCA-3′) and 806 R (5′-GGACTACHVGGGTWTCTAAT-3′) [77], and the fungal ITS-1 region with the primers ITS1F (5′-CTTGGTCAT TTAGAGGAAGTAA-3′) and ITS2R (5’-GCTGCGTTCTTCATC GATGC-3′) [78]. Library construction and Illumina MiSeq sequencing were performed at Majorbio Biopharm Technology Co., Ltd. (Shanghai, China). The obtained raw sequence data were quality filtered and processed using the software system QIIME v1.9.0. Paired-end reads were assigned to samples based on their unique barcode and were merged using FLASH 1.2 software. Sequences (average quality score < 20), improper primers, and ambiguous bases were discarded before clustering. The UCLUST method was used to define the operational taxonomic units (OTUs) at a level of 97% identity. The effective sequences were clustered into OTUs using UPARSE 7.0 software.

### 4.8. Statistical Analysis

Data were analyzed by ANOVA (one-way analysis of variance) using SPSS 17.0. ANOVA were used to analyze the effect of different microbial agents on silage maize yield, soil biochemical properties, and the indices of bacterial and fungal diversity (statistical significance set at *p* < 0.05). Alpha diversity was analyzed using Shannon and Ace indices. Principal coordinates analysis (PCoA) was carried out by the vegan package in the R Studio (4.2.2) based on the Bray–Curtis distance. Significant differences in the relative abundances of dominant bacteria and fungi at the phylum and genus level were analyzed by Kruskal–Wallis test. Redundancy analysis (RDA) was used to characterize the correlations between microbial community structures and soil biochemical properties by CANOCO 5.0. A Mantel test and partial least squares path modeling (PLS-PM) were used to determine the relationship between microbial community, yield, and soil biochemical properties by the linkET package and plspm, respectively, in R Studio.

## 5. Conclusions

The application of microbial agents can significantly alleviate soil salinity and alkalinity stress, improve soil quality, and increase the yield of silage maize. Meanwhile, microbial agents can regulate the structure of rhizosphere microbial communities, enhance rhizosphere soil enzyme activity, and promote the release of soil nutrients, thereby improving the quality of saline–alkaline soils. Microbial agents significantly increased the relative abundances of beneficial genera such as *Arthrobacter*, *Blastococcus*, *Chaetomidium*, *Alternaria*, *Sarocladium*, *Acremonium*, and *Clonostachys*, while significantly decreasing the relative abundances of potential plant pathogenic fungi like *Gibberella* and *Fusarium* after the application of four different microbial agents, which may play crucial roles in the production of silage maize. Moreover, the microbial communities in the soil treated with agents TF1, TF2, and TF3 exhibited positive correlations with NH_4_^+^-N and AK, while those treated with agent TF4 showed positive correlations with AP, EC, pH, and NPA. Therefore, in order to meet the needs of improving saline–alkaline soil health and increasing crop yield, the selection and application of microbial agents should be carried out rationally.

## Figures and Tables

**Table 1:** Effect of PGPRs on plant growth of silage maize in a saline–alkaline fields.

Treatment	Density (×1000 Plants/hm^2^)	Plant Height (cm)	Fresh Weight (kg/Plant)	Stem Diameter (mm)	Biomass (tons/hm^2^)
CK	47.29 ± 8.38 c	213.58 ± 6.46 c	0.80 ± 0.09 b	15.53 ± 0.44 b	39.56 ± 5.69 d
TF1	58.25 ± 3.09 b	255.15 ± 2.44 a	0.91 ± 0.05 ab	22.64 ± 1.31 a	52.69 ± 2.79 c
TF2	57.37 ± 7.95 b	256.25 ± 5.61 a	0.94 ± 0.05 ab	20.69 ± 1.41 a	54.69 ± 7.42 bc
TF3	57.85 ± 0.60 b	252.60 ± 2.53 a	1.06 ± 0.07 a	20.89 ± 0.68 a	60.94 ± 0.44 b
TF4	67.64 ± 2.08 a	233.05 ± 4.67 b	0.97 ± 0.09 ab	20.20 ± 0.57 a	65.41 ± 1.64 a

Data were expressed as the mean ± SE of three replicates. Different lowercase letters behind the corresponding data in the same column mean a statistically significant difference (*p* < 0.05). CK represents treatment with blank control, TF1 represents treatment with *B. subtilis* NCD-2 + *B. amyloliquefaciens* Ba-1, TF2 represents treatment with *B. subtilis* HMB26553 and *B. amyloliquefaciens* PHODG36, TF3 represents treatment with *B. subtilis* WHN-121 and *B. amyloliquefaciens* G35, and TF4 represents treatment with *B. velezensis* B31.

**Table 2:** Effects of PGPRs on soil chemical properties, nutrient content, and enzyme activities.

Index	CK	TF1	TF2	TF3	TF4
OM (%)	1.77 ± 0.21 b	1.93 ± 0.13 b	1.81 ± 0.20 b	2.10 ± 0.22 ab	2.30 ± 0.09 a
NO_3_^−^-N (μg/g)	4.44 ± 0.74 c	5.38 ± 1.09 b	7.34 ± 0.78 a	5.46 ± 0.78 b	5.89 ± 0.36 b
NH_4_^+^-N (μg/g)	53.35 ± 7.34 d	63.41 ± 3.96 c	85.60 ± 5.29 b	95.92 ± 3.20 a	80.34 ± 2.34 b
AK (mg/kg)	937.56 ± 3.90 c	1006.85 ± 9.09 b	1163.77 ± 15.22 a	1116.81 ± 34.02 a	1018.02 ± 8.50 b
AP (μg/g)	168.98 ± 3.70 a	31.43 ± 3.89 c	43.17 ± 2.94 b	41.71 ± 7.77 b	19.19 ± 3.06 d
pH	7.44 ± 0.01 a	7.47 ± 0.05 a	7.09 ± 0.02 c	7.22 ± 0.03 b	7.47 ± 0.01 a
EC (μS/cm)	466.67 ± 0.58 a	313.00 ± 2.00 d	281.33 ± 0.58 e	385.33 ± 2.08 b	343.00 ± 1.00 c
ChtA (U/g)	70.42 ± 1.17 c	75.03 ± 1.97 b	86.67 ± 4.91 a	76.64 ± 1.99 b	82.45 ± 1.78 a
UA (U/g)	2916.10 ± 11.51 d	3165.22 ± 7.85 c	3437.84 ± 16.32 b	3494.24 ± 23.03 b	3682.26 ± 16.92 a
NPA (U/g)	2088.78 ± 45.43 a	1809.46 ± 12.14 d	2006.63 ± 10.12 b	1951.95 ± 11.52 c	1918.76 ± 34.95 c
INVA (U/g)	45.96 ± 1.82 a	48.67 ± 3.56 a	50.28 ± 4.17 a	49.86 ± 2.66 a	49.82 ± 0.94 a

Data were expressed as the mean ± SE of three replicates. Different lowercase letters behind the corresponding data in the same column mean a statistically significant difference (*p* < 0.05). CK represents treatment with blank control, TF1 represents treatment with *B. subtilis* NCD-2 + *B. amyloliquefaciens* Ba-1, TF2 represents treatment with *B. subtilis* HMB26553 and *B. amyloliquefaciens* PHODG36, TF3 represents treatment with *B. subtilis* WHN-121 and *B. amyloliquefaciens* G35, and TF4 represents treatment with *B. velezensis* B31. ChtA, UA, NPA, and INVA represent chitinase activity, urease activity, neutral phosphatase activity, and invertase activity, respectively.

## Data Availability

Raw data of 16S rRNA gene and ITS gene obtained from all samples are accessible via NCBI SRA database under accession numbers were PRJNA1286956 and PRJNA1286967, respectively.
